# GNB3 and CREB1 gene polymorphisms combined with negative life events increase susceptibility to major depression in a Chinese Han population

**DOI:** 10.1371/journal.pone.0170994

**Published:** 2017-02-22

**Authors:** Jingsong Ma, Lin Wang, Yanjie Yang, Zhengxue Qiao, Deyu Fang, Xiaohui Qiu, Xiuxian Yang, Xiongzhao Zhu, Jincai He, Hui Pan, Bo Ban, Yan Zhao, Hong Sui

**Affiliations:** 1 Psychology Department of the Public Health Institute of Harbin Medical University, Harbin, China; 2 Northwestern University Feinberg School of Medicine, Evanston, Illinois, United States of America; 3 Medical Psychological Institute of the Second Xiangya Hospital of Central South University, Changsha, China; 4 The First Affiliated Hospital of Wenzhou Medical University, Wenzhou, China; 5 Peking union Medical College Hospital, Beijing, China; 6 Affiliated Hosptial of Jining Medical University, Jining, China; 7 The Second Affiliated Hosptial of Harbin Medical University, Harbin, China; Xi'an Jiaotong University School of Medicine, CHINA

## Abstract

**Background:**

Major depression (MD) is caused by a combination of genetic and environmental factors. In this study we investigated the interaction of variations in the G-protein beta 3 subunit (GNB3) and cAMP response element binding protein 1 (CREB1) genes with negative life events in the pathogenesis of MD. One GNB3 polymorphism (rs5443) and four CREB1 polymorphisms (rs2253206, rs2551941, rs6740584, rs11904814) were investigated based on known associations with MD.

**Methods:**

512 patients with MD and 513 control subjects were genotyped. The frequency and severity of negative life events were measured by the Life Events Scale (LES). Gene-environment interactions (G×E) were assessed using the generalized multifactor dimensionality reduction (GMDR) method.

**Results:**

Differences in GNB3 rs5443 allele frequencies and genotype distributions were observed between MD patients and controls. Significant G×E interactions were detected between negative life events and genotypic variation of all five single nucleotide polymorphisms (SNPs). Individuals carrying the T^-^ allele of rs5443 (CC), A^-^ allele of rs2253206 (GG), T^-^ allele of rs2551941 (AA), C^-^ allele of rs6740584 (TT) or G^-^ allele of rs11904814 (TT) conferred susceptibility to MD in subjects only exposed to high-negative life events. However, individuals with the T^+^ allele of rs5443 (CT, TT) were susceptible to MD when exposed to low negative life events.

**Conclusions:**

Interactions between GNB3, CREB1 and negative life events were revealed. Further evidence is provided about the role of the environment in genetic vulnerability to MD.

## Introduction

Major depression (MD) is a complex and debilitating psychiatric disorder that causes significant economic burden. Around 5.9% of the Chinese population suffers from a depressive syndrome [1]. The causes of MD are not well defined, but both genetic predisposition and environmental factors play important roles in the pathogenesis of the illness [2].

The development of MD is a multistep process involving the accumulation of multiple genetic changes and concomitant exposure to environment influences such as positive and negative life events. General population-based studies have demonstrated that the genetic predisposition to depression has a 38% heritability, suggesting a contribution from non-genetic factors [3]. It is well known that individuals react differently when exposed to environmental factors [4,5] and these factors, particularly negative life events, are very likely to contribute to the onset of MD [6,7]. It is likely that MD is not attributed solely to genetic or environmental factors. The researches about gene-environment interactions provide a potential pathway to understand how genetic difference with exposure to environmental stress will result in psychopathology. [8].

Guanine nucleotide binding proteins (G-proteins) are key regulators of cellular responses through the cyclic adenosine monophosphate (cAMP) pathways. The abnormal expression and function of G-proteins are closely related to the pathophysiology of a variety of mental illnesses, including MD [9]. G-proteins contain multiple subunits, each encoded by several isoforms. A functional polymorphism (C825T or rs5443) of the G-protein beta3 subunit (GNB3) located on chromosome 12p13.31 has been associated with increased signal transduction and ion transport activity [10], the risk of MD [11], and antidepressant treatment responses [12].

cAMP response element binding protein 1 (CREB1) has also been shown to play an important role in the response of MD patients to antidepressants [13,14]. It is a member of the leucine zipper family of DNA-binding proteins [15] and is activated by neuronal growth factors and stress signals [16]. CREB1 is 69 kb in length and has been mapped to 2q32.3–34 [17]. Li et al. reported an association of CREB1 polymorphisms with a decreased hippocampal volume and diminished activation of the left hippocampus in bipolar disorder patients from Europe [18]. Genetic variations in CREB1 have been confirmed to significantly contribute to mood disorder and MD [19,20].

Evidence suggests that the cAMP signal transduction pathway is a target for antidepressants. After antidepressant treatment, the activation of monoamine receptors results in the generation of cAMP through adenylyl cyclase stimulation by G-proteins [21,22]. The increase in cAMP leads to phosphorylation of the CREB1 transcription factor [23].

Although an increasing body of studies indicated the existence of association between MD and single nucleotide polymorphisms (SNPs) in the GNB3 and CREB1 genes, the findings were not consistent [24,25]. These studies have produced inconsistent results, mainly because they did not consider the interaction of genetic and environmental factors. In the present study, we analyzed the association of GNB3 (rs5443) and CREB1 (rs2253206, rs2551941, rs6740584, rs11904814) SNPs with MD and evaluated how environmental factors contributed to the incidence of MD.

## Materials and methods

### Subjects and clinical assessments

All subjects gave written informed consent to participate and the research was admitted by the Ethics Committee for Medicine of Harbin Medical University, China.

512 patients with MD and 513 control subjects without history of neuropsychiatric disorders were recruited between September 2013 and March 2015. Participates in two groups were all Han Chinese and lived in the north of China. To make a MD diagnosis, participants were interviewed by at least two trained psychiatrists using the Structured Clinical Interview for DSM-IV (SCID-I) [26]. The inter-rater reliability kappa value of SCID was 0.82. The study only included the patients whose minimum scores of the 24-item Hamilton Rating Scale for Depression (HAMD) were 21. Patients without receiving any psychotropic medication in the four weeks of assessment were included. We excluded the patients in case of the following conditions, brain organic mental disorders, other mental disease, and family history of genetic disease, mental retardation, dementia or physical disease. We also excluded the patients who provided insufficient information and accepted recent transfusion therapy. 513 unrelated controls were selected among the healthy individuals visiting the same hospital for physical examination and matched with cases in age (± 3 years). Their families had no substance dependent member, genetic diseases or interracial marriage in three generations.

### Assessment of negative life events

To measure negative life events, we used the Life Events Scale (LES) which was developed by Desen Yang and Yalin Zhang and has been validated in Chinese population. The LES is consisted of 48 items with three dimensions named family life (28 items), work (13 items) and other aspects (7 items), separately [27]. Negative life events included social housing, relationship and social difficulties, serious illness, unemployment and financial crises, relationship breakdowns. A total life events score was calculated from the positive and negative life events determined by the interviewers. There were four aspects of the event being considered in the scale: time of occurrence (absent = 1, more than one year ago = 2, within the past year = 3, chronic = 4), influence on mood (absent = 1, mild = 2, moderate = 3, severe = 4, extreme = 5), duration of influence (≤3 months = 1, 3–6 months = 2, 6–12 months = 3, >12 months = 4) and character (good = 1, bad = 2). We used the 75% percentile (a score of 6) as a cutoff value for high- and low- level negative life events.

### DNA extraction and genotyping

We used the AxyPrep Blood Genomic DNA Miniprep Kit (Axygen, Union City, USA) to get the genomic DNA from 250μl EDTA-anticoagulated venous blood samples. Five SNPs of the GNB3 and CREB1 genes were genotyped: GNB3 (rs5443), CREB1 (rs2253206, rs2551941, rs6740584, rs11904814). We used Primer 5.0 software to design the primers for PCR amplification and used the BLAST of the National Center for Biotechnology Information to check the specificity of each potential primer. DNA samples were genotyped using the 5ʹ nuclease assay with a 7900HT Fast Real-Time PCR System (Applied Biosystems, Foster City, CA, USA). The SNP genotype of each tested sample was determined by computer software and was confirmed manually. Table 1 reports the primer sequences and lengths of the PCR products.

**Table 1:** Primer sequences and length of PCR products.

Gene	SNP ID	Polymorphisms	Primer sequence (5’→3’)	Length of products (bp)
GNB3	rs5443	C/T	F: 5’-CCAATGGAGAGGCCATCTGCA-3’	250
			R: 5’-CTTCCAGCTGAGGAAGCAGCA-3’	
CREB1	rs2253206	A/G	F: 5’-TTCTAGACATTGTGCTGTTGC-3’	228
			R: 5’-TCCCTACTGTAGGCTCTCACT-3’	
	rs2551941	A/T	F: 5’-GCAAGAAAGGGAGGTCTTCGA-3’	252
			R: 5’-CCTGGCCTCTTCCGTCCTGTA-3’	
	rs6740584	C/T	F: 5’-TCCAAACCATGAGTCTCAACT-3’	224
			R: 5’-TTAGGTCTGTATTTCTCAACT-3’	
	rs11904814	G/T	F: 5’-GTGTGAAATCATCAGTTGGAT-3’	225
			R: 5’-TAAGAGGAGCATATGGTTAGG-3’	

### Statistical analysis

We calculated the Hardy-Weinberg equilibrium (HWE) for the genotypic distribution of each SNP using the chi-square goodness-of-fit test. The χ^2^ test was used to compare the frequencies of individuals SNP genotypes and allele between patient group and control group. A P value of < 0.05 (two-tailed) was considered to be statistically significant. We used SPSS package (version 20.0 for Windows) for statistical analyses.

The generalized multifactor dimensionality reduction (GMDR) software (version 0.7) was used to analyze gene-environment interactions, which used cross-validation (CV) to classify and predict disease risk. We defined 10-fold CV and set the threshold ratio at 1.0 in the configuration file. The analysis was run ten times using ten different random number seeds, the results were averaged to avoid faked outcomes because of chance divisions of the data. We selected the model with combination of loci and/ or discrete environmental factors which maximize the CV consistency and minimize the prediction error (PE). When the upper-tail Monte Carlo p-value got by the permutation test was ≤0.05, the null hypothesis was rejected.

The OR values (with 95% CI) of risk factors selected by GMDR analysis were calculated using SPSS for windows (version 20.0) to validate the GMDR results. The dominant models were the only ones to be analyzed in order to narrow down the number of possible combinations and the p-value for multiple comparisons was also corrected using the Bonferroni method.

## Results

### Demographic and clinical data on study subjects

Table 2 reports the general demographic and clinical information of patients and controls. The mean ages for cases (male/female, 145/367) and controls (male/female, 187/326) were 42.97 and 42.50, separately. A significant difference in gender distribution was detected between two groups (P = 0.01). Mean negative LES scores for cases and controls were 10.39 and 3.23, separately. Negative LES scores between cases and controls existed statistically differences (P < 0.00). Furthermore, mean HAMD scores were 30.95 in cases.

**Table 2:** Characteristics of study participants.

Variable	MD (n = 512)	Controls (n = 513)	χ^2^ / t	P-value
Age (mean ± SD)	42.97±12.41	42.50±8.68	0.69	0.49
Gender (males/females)	145/367	187/326	7.74	**0.01**
Negative LES score	10.39±19.69	3.23±10.64	7.24	**< 0.00**
HAMD score	30.95±5.84			

### Single nucleotide polymorphism association analyses

The genotypic distributions of all five polymorphisms conformed to Hardy-Weinberg equilibrium for both patients and controls. The Table 3 reports the genotype and allele distributions for the five SNPs in MD patients and control subjects. There was a significant difference in the genotypic distribution between patients and controls for rs5443 (χ^2^ = 45.27, P < 0.00) and in allelic association (χ^2^ = 8.76, P = 0.00, OR = 0.77, 95% CI: 0.65–0.92). But there was no significant association between CREB1 SNPs and MD (rs2253206, allelic: P = 0.79, genotypic: P = 0.89; rs2551941, allelic: P = 0.54, genotypic: P = 0.83; rs6740584, allelic: P = 0.94, genotypic: P = 0.94; rs11904814, allelic: P = 0.86, genotypic: P = 0.98).

**Table 3:** Distributions of genotypes and alleles for study participants.

Gene		MD (n = 512)	Controls (n = 513)	P-value	OR (95% CI)
GNB3	rs5443				
	CC	88 (17.19)	170 (33.14)	**< 0.00**	
	CT	299 (58.40)	203 (39.57)		
	TT	125 (24.41)	140 (27.29)		
	C	475 (46.39)	543 (52.92)	**0.00**	0.77 (0.65–0.92)
	T	549 (53.61)	483 (47.08)		
CREB1	rs2253206				
	AA	81 (15.82)	82 (15.98)	0.89	
	AG	244 (47.66)	237 (46.20)		
	GG	187 (36.52)	194 (37.82)		
	A	406 (39.65)	401 (39.08)	0.79	1.02 (0.86–1.22)
	G	618 (60.35)	625 (60.92)		
	rs2551941				
	AA	105 (20.51)	98 (19.10)	0.83	
	AT	237 (46.29)	238 (46.40)		
	TT	170 (33.20)	177 (34.50)		
	A	447 (43.65)	434 (42.30)	0.54	1.06 (0.89–1.26)
	T	577 (56.35)	592 (57.70)		
	rs6740584				
	CC	191 (37.30)	195 (38.01)	0.94	
	CT	244 (47.66)	239 (46.59)		
	TT	77 (15.04)	79 (15.40)		
	C	626 (61.13)	629 (61.31)	0.94	0.99 (0.83–1.19)
	T	398 (38.87)	397 (38.69)		
	rs11904814				
	GG	80 (15.63)	78 (15.21)	0.98	
	GT	245 (47.85)	246 (47.95)		
	TT	187 (36.52)	189 (36.84)		
	G	405 (39.55)	402 (39.18)	0.86	1.02 (0.85–1.21)
	T	619 (60.45)	624 (60.82)		

### Gene-environment interaction

We used GMDR method to analyze the gene-environment interaction. In order to simplify the meaning of biological information, we set the number of interacting factors at either 2 or 6. The maximum values for CV and PE were yielded through the most significant model revealed by GMDR analysis. As shown in Table 4, the interaction between the rs5443 and negative life events had a CV consistency of 10 and PE of 0.36, which was considered the best of the two factors; the interaction between the rs5443-rs2253206-rs2551941-rs6740584-rs11904814 combination and negative life events had a CV consistency of 10 and PE of 0.31, which was considered the best of the six factors. The result of permutation test suggested that the prediction error of the interaction was significant at the 0.00 level.

**Table 4:** GMDR analysis of gene-environmental interactions in MD patients and controls.

NO. of factors considered	Best models	Prediction error	Cross-validation consistency	P-value
2	rs5443, Negative life event	0.36	10/10	**0.00**
6	rs5443, rs2253206, rs2551941, rs6740584, rs11904814, Negative life event	0.31	10/10	**0.00**

P-values were calculated by permuting the cases and controls 1000 times.

The OR (with 95% CI) values were calculated to assess the set of risk factors selected by the GMDR method. The Table 5 showed that the analyses performed assuming a single contribution of each gene-environment interaction to MD risk suggested an OR value of 6.63 (95% CI 3.71–11.84) in individuals carrying the T- allele (CC) of rs5443 associated with high-negative life events. In individuals with the other genotypes of rs5443, low-negative life events gave OR values of 3.35 (95% CI 2.24–5.01). Furthermore, among individuals carrying the A^-^ allele (GG) of rs2253206, T^-^ allele (AA) of rs2551941, C^-^ allele (TT) of rs6740584 or G^-^ allele (TT) of rs11904814, highly-negative life events yielded OR values of 7.45 (95% CI 3.84–14.45), 3.89 (95% CI 2.03–7.48), 3.72 (95% CI 1.78–7.76) and 7.26 (95% CI 3.74–14.08), indicative of a higher susceptibility to MD.

**Table 5:** Interaction of GNB3 and CREB1 genetic polymorphisms with negative life events.

Variable	MD (n = 512)	Controls (n = 513)	OR^a^ (95% CI)	P-value
rs5443				
T^-^ and LN	36	139	1	
T^-^ and HN	52	31	6.63 (3.71–11.84)	**< 0.00**
T^+^ and LN	274	315	3.35 (2.24–5.01)	**< 0.00**
T^+^ and HN	150	28	0.96 (0.47–1.99)	0.92
rs2253206				
A^-^ and LN	126	182	1	
A^-^ and HN	61	12	7.45 (3.84–14.45)	**< 0.00**
A^+^ and LN	184	272	0.96 (0.71–1.29)	0.79
A^+^ and HN	141	47	0.62 (0.29–1.32)	0.22
rs2551941				
T^-^ and LN	58	81	1	
T^-^ and HN	47	17	3.89 (2.03–7.48)	**< 0.00**
T^+^ and LN	252	373	0.95 (0.65–1.38)	0.78
T^+^ and HN	155	42	1.45 (0.68–3.09)	0.33
rs6740584				
C^-^ and LN	43	65	1	
C^-^ and HN	34	14	3.72 (1.78–7.76)	**< 0.00**
C^+^ and LN	267	389	1.04 (0.68–1.58)	0.86
C^+^ and HN	168	45	1.51 (0.66–3.44)	0.33
rs11904814				
G^-^ and LN	126	177	1	
G^-^ and HN	61	12	7.26 (3.74–14.08)	**< 0.00**
G^+^ and LN	184	277	0.92 (0.68–1.24)	0.58
G^+^ and HN	141	47	0.64 (0.30–1.38)	0.26

LN: low-negative life events; HN: high-negative life events.

OR: odds ratio; CI: confidence interval.

^a^: Adjusted for age and gender.

## Discussion

In the present study, we have defined allelic variations in the GNB3 and CREB1 genes that confer susceptibility to MD in combination with negative life events in Chinese Han individuals. The role of intracellular pathways and signal transduction cascades in both the pathophysiology and treatment of depression have been known since the 1980s [28]. In recent years, members of the cAMP cascade have been identified as targets for antidepressants. G-proteins play a crucial role in the integration, regulation and amplification of downstream signal cascades including the cAMP system, phosphatidylinositols and ion channels [29]. Altered expression and function of G-proteins has been implicated in a number of diseases, including MD [30].

Evidences shown that C825T (rs5443), a SNP located in the coding exon region of GNB3, was associated with increased risk of MD. Zill et al. reported an association between rs5443 and MD, and the frequency of the T allele was significantly higher in MD patients than in healthy controls [14]. Subsequently, Lee et al. identified rs5443 was associated with the symptomatology and treatment responses of MD in the Korean population [15]. Our study has confirmed that rs5443 was possible susceptibility loci for MD onset. This is in agreement with previous findings, although one study in the Japanese population reported that rs5443 does not play a major role in susceptibility to MD [24].

On the other hand, the association between CREB1 polymoprhisms and MD is also involved in the present study. The CREB1 has been implicated in the pathophysiology of depression and in the response to antidepressant treatment [16,17]. Zubenko et al. identified an association of rs2253206 with mood disorder and MD in females [22], and Utge et al. reported an association between rs11904814 and MD in males [23]. However, such an association was not observed in our study. The discrepancies between these studies may be explained by differences in sample size, ethnicity and disease characteristics, but we believe that the lack of investigation into a potential history of stressful life events may be another significant source of discrepancy, based on our findings that variations in CREB1 contribute to MD pathogenesis in combination with negative life events.

Large genetic influences on disease are rapidly eliminated by evolution, therefore highly prevalent diseases such as mental disorders are usually associated with multiple genes, each making a minor contribution [31]. Allelic variants also interact with other variants [32] and with environmental factors to influence the risk of psychiatric disease [33]. Clinical studies have found that negative life events do influence the risk and severity of MD [6,7]. However, individuals vary greatly in their behavioral response to life events. These variations can be attributed to differences in genetic predisposition and life experience [34] and these factors interact to influence the neural systems involved in MD.

In this study, negative life events experienced include death and illness of relatives, marriage problems, unemployment, economic difficulties, and lost of social relationships. Life stress is associated with structural and functional changes in the brain, and these changes are modulated by genetic factors [35]. Significant interactions between 5-HTTPR and BDNF genes and stressful life events have been reported in patients with depression [36–38]. Moreover, SNPs in the FKBP5 gene increase the risk of psychotic symptoms in young adults in combination with childhood maltreatment [39]. Our results shown an interaction between GNB3 (rs5443) and CREB1 (rs2253206, rs2551941, rs6740584, rs11904814) SNPs genotypes and negative life events in MD. Individuals with the CC genotype of rs5443, GG genotype of rs2253206, AA genotype of rs2551941, TT genotype of rs6740584, or TT genotype of rs11904814 were susceptible to MD only when exposed to high-negative life events, while the other genotypes of rs5443 conferred susceptibility to MD in subjects exposed to low-negative life events. Further evidence for an interaction between GNB3, CREB1 and environment in psychiatric illness risk is provided in our study. Animal studies suggested that CREB1 has an important role in MD, and modulates the behavioral response to adverse environments and episodes [40]. In addition, Wang et al. [41] reported a significant interaction between CREB1 polymoprhisms and family harmony and childhood trauma in MD patients.

There are some limitations to the present study. First, negative life events were assessed by subjective interpretation. Second, there were only including subjects with Chinese Han origin from the north of China in the study and the sample sizes were relatively small. So our findings can not be generalized to other populations. Nonetheless, a homogenous population is more advantageous to reveal specific gene-disease associations and may be essential for the research with gene-environment effects.

## Conclusions

In summary, we have provided evidence that GNB3 and CREB1 variations interact with environmental factors to increase the risk of MD. As the candidate SNPs examined have been shown to regulate GNB3 and CREB1 expression, it seems likely that life events modulate this influence on expression. Our study should now be replicated in larger populations with different ethnicities. It would also be interesting to investigate the effect of these SNP-environment interactions on the cAMP system.

## Supporting information

S1 Dataset Dataset of the subsamples. (SAV)
